# Irradiation with Polychromatic Incoherent Low Energy Radiation of Human Peripheral Blood Mononuclear Cells In Vitro: Effects on Cytokine Production

**DOI:** 10.3390/ijerph17041233

**Published:** 2020-02-14

**Authors:** Francesca Maria Salmeri, Lucia Denaro, Elisa Ruello, Giuseppe Acri, Sergio Gurgone, Carlo Sansotta, Barbara Testagrossa

**Affiliations:** 1 Department of BIOMORF, University of Messina, Via Consolare Valeria, 98125 Messina, Italy; fmsalmeri@unime.it (F.M.S.); ldenaro@unime.it (L.D.); eruello@unime.it (E.R.); sansotta@unime.it (C.S.); btestagrossa@unime.it (B.T.); 2 Department of MIFT, University of Messina, Viale F. Stagno d’Alcontres, 98166 Messina, Italy; sgurgone@unime.it

**Keywords:** PILER, PBMCs, Cytokines

## Abstract

(1) Background: Physical stimuli may activate peripheral blood mononuclear cells (PBMCs) to secrete cytokines, which may favor pro-inflammatory responses or trigger reparative phenomena. The purpose of this study is to evaluate the action of Polarized Polychromatic Incoherent Low Energy Radiation (PILER) on human in vitro PBMCs, by detection of the possible effects on cytokine production; (2) Methods: isolated PBMCs were irradiated with a PILER lamp at different exposure times, at a distance of 10 cm, before incubation. The supernatants were collected after 24 h and 48 h and cytokines evaluated by ELISA; (3) Results: Our results showed a decrease in the levels of pro-inflammatory IL-12p70, IL-17A, IFN-γ, and TNF-α cytokines, whereas IL-10 and TGF-β1 with regulatory activity increased; (4) Conclusions: PILER irradiation affected the cytokine production by isolated PBMCs driving the immune response toward an anti-inflammatory/reparative profile.

## 1. Introduction

Many studies reporting the effects of light therapy on humans, in different pathological conditions, concern the irradiation by the application of ultraviolet (UV) lamp [1,2], infrared (IR) laser [3,4], Light Emitting Diode (LED) [5,6], and Polarized Polychromatic Incoherent Low Energy Radiation (PILER) [7,8,9,10,11]. All these sources showed light therapy exerts reparative capacity by modulating the production of cytokines and other soluble factors, and their main targets are fibroblasts from the skin [7].

We focused on the possible effects of PILER irradiation. In a previous work [12], we studied the phototreatment of chronic radiodermitis by a physician by using a PILER lamp. We observed an improvement in the irradiated skin and functionality of the hand after two months. A clear amelioration of symptoms with rosy skin of the fingers and the disappearance of ulcers and bleeding, as well as the repair of the underlying connective tissue with a sharp reduction of onychodystrophy, occurred after one year [12].

Despite clinical evidence, nevertheless, the possible cellular and molecular targets of light sources are poorly understood. Some studies observed that UV [13,14], IR [4,15], and LED [15,16] sources provoked variations of immune cell functionality and the production of cytokines and other soluble factors.

Only a few experiments have been performed using PILER radiation to analyze variations in immunological parameters in different physiological or pathological conditions, such as number of leukocytes, phagocytic capability of neutrophils and macrophages, blood plasma immunoglobulin concentrations, lymphocyte subpopulations, NK and CD8^+^T cells cytotoxic activity, and blood serum cytokine levels [17,18,19,20,21,22].

Considering our previous clinical experience and the presence of data from the literature, the aim of this work is to study the action of PILER irradiation, at different exposure times, on human peripheral blood mononuclear cells (PBMCs) by detection of the possible effects on the cytokine production. 

## 2. Materials and Methods 

### 2.1. Subjects

The work was carried out at the Department of BIOMORF, University of Messina, Italy, from May 2018 to June 2019. Twenty apparently healthy volunteers of our staff were enrolled in the study. The inclusion criteria are reported in the itemized list below:

- Age 20 to 45 (mean age of 32.7 ± 8.1, M:F = 9:1),
- Hematologic parameters within the range of physiological values,
- Leukocytes ranging from 6000/μL to 9000/μL,
- Lymphocytes ranging from 1800/μL to 2600/μL,
- Monocytes ranging from 270/μL to 420/μL.

The exclusion criteria included:

- History of UV exposure for at least 6 months before the study,
- History of photosensitivity,
- History of acute or chronic inflammatory diseases,
- History of auto-immune, cardiovascular, metabolic, endocrine, and oncological diseases,
- Oral intake of medications (e.g., steroidal and/or non-steroidal anti-inflammatory agents, photosensitizing agents),
- Treatment with hormone therapy,
- Treatment for cancer (chemotherapy, immunotherapy, biological therapy, radiation),
- Pregnancy.

All the involved volunteers could understand the study design and objectives. Before participating in our study, they signed an informed consent form allowing us to use their biological fluids and cells.

### 2.2. PILER Source

**Figure 1:**
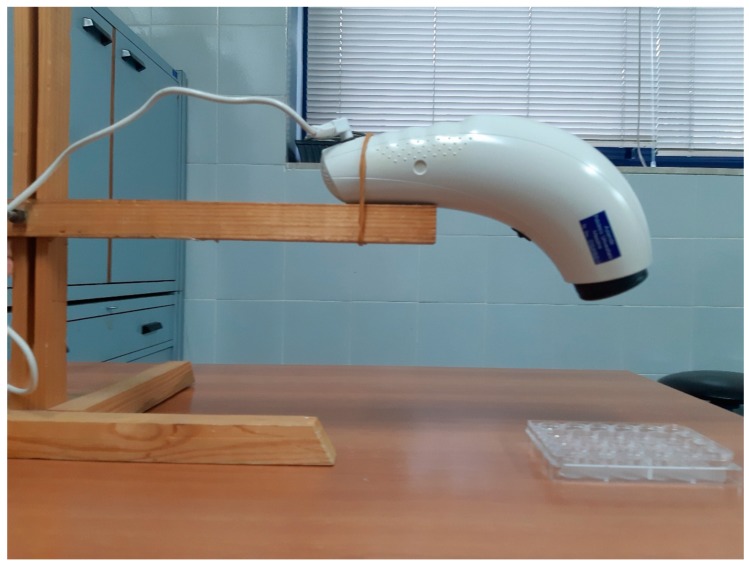
Bioptron lamp.

A PILER Bioptron lamp (Zepter International, Switzerland) was used to irradiate PBMCs. The lamp emanated a linearly polarized incoherent light in the visible-infrared part of A and B (400 nm ≤ λ ≤ 2000 nm), with power density of 40 mW/cm^2^, radiation intensity of 2.4 J/cm^2^, penetration depth of 2.5 cm, lamp power of 20 W. The lamp (Figure 1) was positioned at a 10 cm distance from the plane of the uncovered plates containing cells. Major details about cell treatment are thoroughly explained in Section 2.3.2.

### 2.3. Cells

#### 2.3.1. PBMC Collection and Preparation

Blood was obtained by venipuncture using heparin as an anticoagulant and PBMCs were isolated at room temperature by density gradient centrifugation at 400× *g* of the venous samples on a polysucrose and sodium diatrizoate solution (Histopaque-1077, Sigma, St. Louis, MO, USA) for 30 min, according to the technique of Böyum [23]. The PBMCs were collected at the interface, washed twice in phosphate-buffered saline (PBS), counted, and suspended again at a concentration of 1 × 10^7^/mL in a culture medium of PBS supplemented with 10% heat-inactivated fetal bovine serum (Sigma, St. Louis, MO, USA), 2 mM glutamine (Sigma, St. Louis, MO, USA), 100 U/mL penicillin–streptomycin (Sigma, St. Louis, MO, USA), 10 mM HEPES, and 50 M mercaptoethanol (Sigma, St. Louis, MO, USA).

#### 2.3.2. PBMC Treatment

Samples of 100 μL containing 1 × 10^6^ cells of PBMCs of healthy subjects were placed in uncovered polystyrene 24-well plates (diameter 15 mm) and irradiated with the PILER lamp for different exposure times (6, 12, 18, 24, and 30 min, respectively). The cells, supplemented with culture media to a final volume of 1 mL, were then incubated in a 5% CO_2_ incubator at 37 °C, in a humidified atmosphere, for 24 h and 48 h. Unirradiated PBMCs of the same subjects were incubated in the same conditions and used as controls. No mitogen was used to stimulate PBMCs. All treatments were performed in a sterile environment to avoid any accidental contamination and improper stimulation of the cells. At the end of incubation, the viability of PBMCs was >95%, as assessed by the Trypan blue exclusion method. Cell suspensions were centrifuged at 400× *g* for 15 min and the supernatants harvested, aliquoted, and stored at −80 °C until being used for cytokine determination by enzyme-linked immunosorbent assay (ELISA).

### 2.4. Determination of Cytokines by ELISA

**Figure 2:**
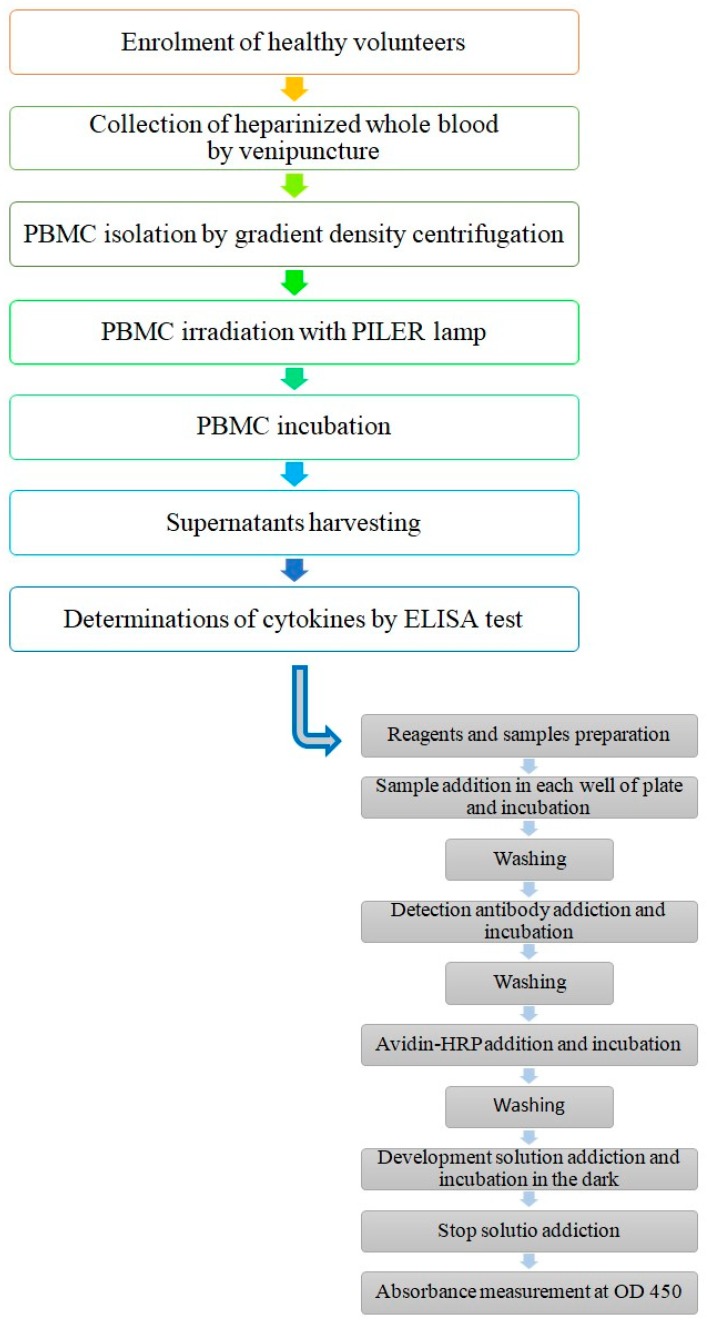
Flow chart of major steps followed in this study.

Cytokine determinations were performed by standard ELISA, using a multi-analyte ELISArray kit (MEH-003A, Qiagen, Germany) that simultaneously detects the level of multiple cytokines using the conventional sandwich-based ELISA technique. In particular, we evaluated the presence of IL-2, IL-4, IL-5, IL-6, IL-10, IL-12p70, IL-13, IL-17A, IFN-γ, TNF-α, IL-23, and TGF-β1, according to the manufacturer’s instructions. We analyzed the supernatants of two independent experiments. The standard curve provided in the kit was used to calculate the cytokine concentrations, expressed as pg/mL. Briefly, triplicate aliquots of each sample, including positive and negative control specimens, and experimental specimens, were placed in 96-precoated microtiter plates. During the first incubation, capture antibodies bound their specific cytokine antigen. A new washing was performed to remove the unbound cytokines and specific biotinylated detector antibodies were added to the wells to capture the appropriate cytokine. After washing again to remove the unbound reagents, an enzyme (avidin–horseradish–peroxidase-conjugated) was added to the wells. After incubation and washing to remove the entire unbound enzyme, a colorimetric substrate solution was added and finally, color development was stopped by the addition of H_2_SO_4_. The colored product was directly proportional to the concentration of the specific cytokine present in the samples. The absorbance of each well was measured by means of a spectrophotometer (BioTek Instruments, Inc., VT, USA) using 450 nm as a detection wavelength (Figure 2).

### 2.5. Statistical Analysis

To validate the obtained results, we performed a three-step statistical analysis by using MATLAB software (MathWorks, Inc., MA, USA). First, we performed a Lilliefors test (using the “lillietest” MATLAB function) to ensure that the collected datasets were following a normal distribution [24]. Then, we performed a Kruskal–Wallis test (using the “kruskalwallis” MATLAB function,) that allows the comparison of different samples with any restriction about the distribution, which can be either normal or one of the many non-normal distributions (including a skewed one) to verify the distribution of datasets [25]. Finally, we performed a multiple comparison based on the Tukey’s range test (using the “multcompare” MATLAB function) to ascertain which groups belong to different populations with respect to the others [26].

## 3. Results

The Lilliefors test showed that not all datasets came from normally distributed populations, and for this reason, we chose the Kruskal–Wallis test instead of an ANOVA test. This showed that the data did not come from the same distribution (*p* < 0.001).

**Figure 3:**
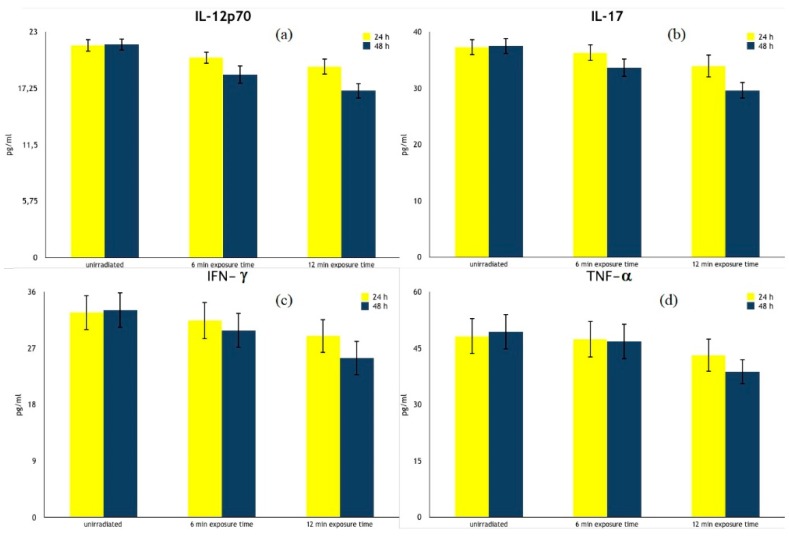
Pro-inflammatory cytokine production by Polarized Polychromatic Incoherent Low Energy Radiation (PILER) exposed and cultured human peripheral blood mononuclear cells (PBMCs). The results are expressed as the means of cytokine levels ± SD of 20 healthy volunteers and are expressed as pg/mL. (**a**) IL-12p70; (**b**) IL-17; (**c**) IFN-γ; (**d**) TNF-α.

With the multiple comparison, we found a statistically significant difference in the production of specific pro-inflammatory cytokines (better detailed in Table 1 and Figure 3). In particular, when cells were irradiated with PILER lamp for 6 min and cultured for 48 h, the levels of IL-12p70 (18.65 ± 0.94 pg/mL) and IL-17A (33.59 ± 1.62 pg/mL) decreased, with respect to the unirradiated PBMCs ones (21.71 ± 0.60 pg/mL and 37.50 ± 1.42 pg/mL, respectively) (*p* < 0.001 in both cases). Levels of pro-inflammatory IFN-γ (29.85 ± 2.80 pg/mL) decreased as well when compared to the unirradiated PBMCs ones (33.11 ± 2.81 pg/mL) although less markedly (*p* < 0.05).

The 12 min exposure time provoked a statistically significant decrease in the production of the same cytokines (*p* < 0.001) for both 24 h- and 48 h-culture times (Table 1 and Figure 3a–c) compared with unirradiated PBMCs. Moreover, we observed a statistically significant decrease in levels of TNF-α for both 24 h- and 48 h-culture times (*p* < 0.05 and *p* < 0.001, respectively) (Figure 3d). The levels of these cytokines did not undergo further changes at 18, 24, and 30 min exposure times (data not shown).

**Figure 4:**
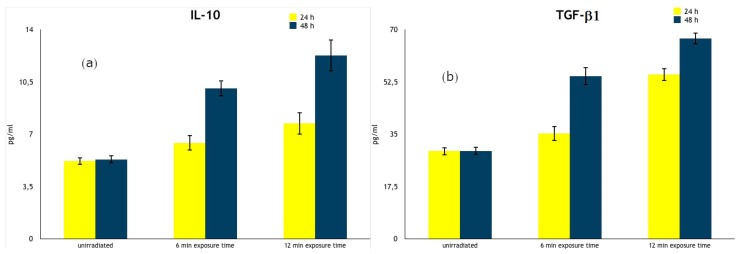
Anti-inflammatory cytokine production by PILER exposed and cultured human PBMCs. The results are expressed as the means of cytokine levels ± SD of 20 healthy volunteers and are expressed as pg/mL. (**a**) IL-10; (**b**) TGF-β1.

IL-10 and TGF-β1 regulatory cytokines behaved in an opposite way (Table 2 and Figure 4). We observed a statistically significant increase in their production by irradiated PBMCs with respect to unirradiated cells. Particularly, levels of IL-10 augmented when cells were exposed for 6 min at both 24 h (6.42 ± 0.52 pg/mL vs. 5.20 ± 0.25 pg/mL, *p* < 0.05) and 48 h (10.06 ± 0.52 pg/mL vs. 5.32 ± 0.27 pg/mL, *p* < 0.001) culture times (Figure 4a). The levels of TGF-β increased at 48 h (54.37 ± 3.02 pg/mL vs. 29.41 ± 1.37 pg/mL, *p* < 0.001) culture time (Figure 4b) only. We also observed a statistically significant increase in the production of these regulatory cytokines following 12 min exposure at both 24 h (*p* < 0.001) and 48 h (*p* < 0.001) culture times. Hence, also in this case, the variations in IL-10 and TGF-β1 levels did not change when PBMCs were exposed to PILER lamp for 18, 24, and 30 min (data not shown).

The concentrations of all other tested cytokines (IL-2, IL-4, IL-5, IL-6, IL-13, and IL-23) did not differ between irradiated and unirradiated PBMCs (data not shown).

## 4. Discussion

The use of light therapy to ameliorate different human pathological conditions is supported by clinical evidence, but targets of their action are under investigation. PILER irradiation as well as UV [13,14], IR [4,15], and LED [15,16] sources may act as non-infectious (“sterile”) agents to activate cell-mediated immune response by modulating the production of cytokines and other soluble factors.

The so-called “mononuclear cells”, blood circulating lymphocytes and monocytes, and their effector subsets, such as effector Th1, Th2, Th17, and Tregs [27], and M1 and M2 macrophages [28], are the main cells of the immune system and produce soluble cytokines after their recruitment and activation in tissues. Cytokines are small proteins, which act as “messengers” of the immune system; they have pleiotropic activity and play a critical role in host defense against foreign antigens [29]. The cytokinic microenvironment can drive the immune response towards inflammatory processes or anti-inflammatory/reparative mechanisms, depending on the cytokine profile prevailing locally.

Pro-inflammatory cytokine profile includes IL-12, TNF-α, IFN-γ, IL-17A, GM-CSF, TNF-α, IL-1α, IL-2, IL-6, and IL-23, whereas IL-4, IL-5, and IL-13 trigger the anti-inflammatory/reparative mechanisms. Finally, IL-10 and TGF-β are regulatory cytokines, which are essential to maintain the balance between pro- and anti-inflammatory responses [29].

In our study, despite the small number of samples, we observed a homogeneous decrease in IL-12p70, IL-17A, IFN-γ, and TNF-α pro-inflammatory cytokines, whereas the regulatory IL-10 and TGF-β1 cytokines increased. IL-10 exerts multiple effects in immuno-regulation and inflammation. This cytokine can block pro-inflammatory transcription factors, leading to the downregulation of the expression of Th1 cytokines, MHC class II, and costimulatory molecules on macrophages. It also enhances B cell survival, proliferation, and antibody production [30]. TGF-β1 is also an anti-inflammatory cytokine known to regulate both cell proliferation and apoptosis, cell differentiation, intercellular adhesion, tissue recycling, angiogenesis, and collagen synthesis. All of them are mechanisms playing a role in wound repair [31].

To study the effect of PILER irradiation on the human immune response, Zhevago et al. [18,19,20] examined the effects of polychromatic visible + polarized IR light (wavelength: 480–3400 nm, radiation intensity 12 J/cm^2^) on the immune system after irradiation of both the sacral area of healthy volunteers and the whole blood sample in vitro. They evaluated the PHA-induced proliferation of peripheral blood lymphocytes, showing an increased lymphocyte proliferation and a fast decrease in the elevated pro-inflammatory cytokines, such as TNF-α, IL-6, IL-12, and IFN-γ. In parallel with a decrease in the pro-inflammatory factor levels, the anti-inflammatory cytokines, such as IL-10, PDGF, and TGF-β1, increased. These changes resulted from transcutaneous photo-modification of a small volume of blood and their fast transfer to the entire pool of circulating blood [19]. These studies are similar to our work, but our experimental conditions are quite different. First, the present study was performed by irradiating the isolated human PBMCs directly without any cover or skin, which may act as a barrier and modify the actual effect of radiation. Second, we did not stimulate PBMCs with any mitogen to detect if a variation of cytokine production occurred spontaneously.

The increase in IL-10 and TGF-β1 might have the effect of directing the immune cells towards an anti-inflammatory/reparative and regulatory response. In this way, the inflammatory processes can be limited, and the reparative phase triggers. Our results may, in part, explain the healing of the wounded area and the improvement in chronic inflammatory processes, previously observed by us [12] and other researches [8,9,10,11,17,18,19,20,21,22] in vivo.

## 5. Conclusions

We showed that PILER irradiation might act as a “sterile” trigger on human in vitro isolated PBMCs, affecting their cytokine production and driving the immune response towards an anti-inflammatory/reparative profile and representing a non-pharmaceutical and non-invasive option for a number of clinical conditions such as the treatment of human wounds.

## Figures and Tables

**Table 1:** Effects of exposure to a Polarized Polychromatic Incoherent Low Energy Radiation (PILER) lamp on the production of the pro-inflammatory cytokines by cultured peripheral blood mononuclear cells (PBMCs).

Cytokines	Time of Culture	Unirradiated PBMCs	6 min Exposure Time	12 min Exposure Time
IL-12p70 (pg/mL)	24 h	21.60 ± 0.66	20.36 ± 0.63	19.45 ± 0.80 ^2^
	48 h	21.71 ± 0.60	18.65 ± 0.94 ^2^	17.00 ± 0.78 ^2^
IL-17 (pg/mL)	24 h	37.26 ± 1.45	36.27 ± 1.47	33.94 ± 2.03 ^2^
	48 h	37.50 ± 1.42	33.59 ± 1.62^2^	29.60 ± 1.45 ^2^
IFN-γ (pg/mL)	24 h	32.70 ± 2.80	31.43 ± 2.99	28.97 ± 2.70 ^1^
	48 h	33.11 ± 2.81	29.85 ± 2.80 ^1^	25.46 ± 2.72 ^2,3^
TNF-α (pg/mL)	24 h	48.21 ± 4.78	47.38 ± 4.83	43.10 ± 4.38 ^1^
	48 h	49.29 ± 4.72	46.73 ± 4.69	38.72 ± 3.32 ^2,4^

Data are the mean ± SD of 20 healthy volunteers and are expressed as pg/ml. ^1^ *p* < 0.05 vs. unirradiated PBMCs, ^2^ *p* < 0.001 vs. unirradiated PBMCs, ^3^ *p* < 0.05 vs. 6’ irradiation time, ^4^ *p* < 0.001 vs. 6’ irradiation time.

**Table 2:** Effects of exposure to PILER lamp on the regulatory cytokines production by cultured PBMCs.

Cytokines	Time of Culture	Unirradiated PBMCs	6 min Exposure Time	12 min Exposure Time
IL-10 (pg/mL)	24 h	5.20 ± 0.25	6.42 ± 0.52 ^1^	7.72 ± 0.73 ^2^
	48 h	5.32 ± 0.27	10.06 ± 0.52 ^2^	12.26 ± 1.07 ^2^
TGF-β1 (pg/mL)	24 h	29.33 ± 1.35	35.27 ± 2.46	54.93 ± 2.19 ^2,3^
	48 h	29.41 ± 1.37	54.37 ± 3.02 ^2^	66.97 ± 1.93 ^2^

Data are the mean ± SD of 20 healthy volunteers and are expressed as pg/mL. ^1^ *p* < 0.05 vs. unirradiated PBMCs, ^2^ *p* < 0.001 vs. unirradiated PBMCs, ^3^ *p* < 0.05 vs. 6’ irradiation time.
